# Bioactive Glass-Inspired Coating for Implants via Plasma Electrolytic Oxidation: A Preclinical Approach to Enhance Bone Repair in Healing-Impaired Conditions Associated with Diabetes

**DOI:** 10.1021/acsomega.5c08289

**Published:** 2025-12-18

**Authors:** Stéfany Barbosa Alves da Cruz, Raphael Cavalcante Costa, Francieli da Silva Flores, Maria Helena R. Borges, Doris Hissako Matsushita, Martinna Bertolini, Nilson Cristino da Cruz, João Gabriel S. Souza, Edilson Ervolino, Valentim A. R. Barão, Leonardo P. Faverani

**Affiliations:** † Department of Diagnosis and Surgery, Dentistry School, 207339 Universidade Estadual Paulista (UNESP), Araçatuba 16015-050, São Paulo, Brazil; ‡ Department of Prosthodontics and Periodontology, Piracicaba Dental School, 28132 Universidade Estadual de Campinas (UNICAMP), Piracicaba 13414-903, São Paulo, Brazil; § School of Dentistry, Alfenas Federal University (UNIFAL-MG), Alfenas 37130-001, Minas Gerais, Brazil; ∥ Department of Basic Science, Dentistry School, 89121 Universidade Estadual Paulista (UNESP), Araçatuba 16015-050, São Paulo, Brazil; ⊥ Department of Periodontics and Preventive Dentistry, 6614 University of Pittsburgh, Pittsburgh 15261, Pennsylvania, United States; $ Laboratory of Technological Plasmas, Engineering College, Universidade Estadual Paulista (UNESP), Sorocaba 18087-180, São Paulo, Brazil; ∇ Department of Periodontology, Dental Research Division, Guarulhos University, Guarulhos 07023-070, São Paulo, Brazil; ○ Department of Oral Diagnosis, Division of Oral and Maxillofacial Surgery, Piracicaba Dental School, 245124 Universidade Estadual de Campinas (UNICAMP), Piracicaba 13414-903, São Paulo, Brazil

## Abstract

Diabetes mellitus is a globally prevalent metabolic disorder that impairs wound healing and bone regeneration, compromising outcomes in implant therapies that rely on osseointegration. Advances in precision medicine and bioengineering have driven the development of functionalized implant surfaces to overcome these limitations. Among them, bioactive glass (BG) coatings have emerged as promising candidates to enhance biological responses. Building upon this rationale, we unveiled the osteoinductive potential of a BG-based coating synthesized via plasma electrolytic oxidation (PEO) and its effects on peri-implant bone regeneration in a diabetic rat model. Titanium implants were treated with PEO using a formulation mimicking BG composition (∼45.0 Si, 24.5 Ca, 24.5 Na, 6.0 P; m/v %), and the resulting coating was characterized. Implants with a sandblasted and acid-etched (SLA) surface served as the control. *In vivo* evaluation was conducted in Wistar rats with streptozotocin-induced diabetes mellitus, followed by tibial implant placement. At 14 and 28 days postimplantation, samples were harvested for histological, immunohistochemical, micro-CT, and histomorphometric analyses. Physicochemical characterization confirmed the synthesis of the PEO-BG coating, which exhibited enhanced surface roughness and wettability compared to SLA controls. A significantly greater area of newly formed bone, increased bone–implant contact, and favorable bone turnover were noted in the PEO-BG group. The expression profiles of BMP-2, RANKL, OPG, and OCN indicated modulation of osteogenic and inflammatory pathways consistent with accelerated bone repair. These findings demonstrate that PEO-BG coating confers robust osteoinductive potential, enhancing peri-implant bone regeneration under compromised diabetic conditions, and highlight its potential for translational application in high-risk populations.

## Graphical abstract

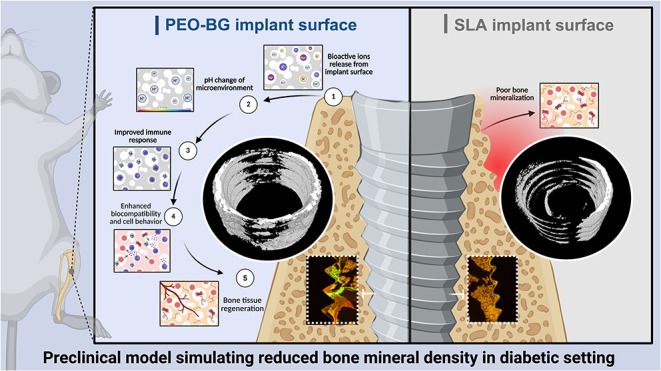

## 1 Introduction

Dental and orthopedic implant success depends on sufficient bone volume and quality, as well as a sustainable healing process after implant placement to ensure proper osseointegration and long-term stability.^1,2^ Following implant insertion, an orchestrated cascade of wound-healing events, regulated by various cell types, initiates bone formation around the implant.^3^ Without complications, the following events will lead to osseointegration of the implant: hemostasis and coagulum formation, granulation tissue development, bone formation, and bone remodeling.^3,4^ However, pathological conditions, such as diabetes mellitus, pose significant challenges for implant integration.^5,6^ Diabetes is a prevalent condition projected to affect more than 1 billion people by 2025.^7^ It is characterized by elevated blood glucose levels resulting from abnormal β-cell function and impaired insulin action,^8^ which also affects wound healing in both soft and hard oral tissues.^9^ Diabetes can negatively impact bone formation by altering the activity of osteoclasts, osteoblasts, and mesenchymal stem cells, processes closely linked to glucose levels.^9^ In fact, diabetes is associated with impaired bone regeneration and persistent inflammation.^10,11^ Systematic reviews have shown that poorly controlled diabetes can compromise dental implant osseointegration, even reducing bone-to-implant contact.^12^ In these cases, conventional implant surfaces may lack the necessary biological stimulation to promote new bone formation, increasing the risk of implant failure.^13,14^

To overcome such challenges in clinical settings, advancements in precision medicine and bioengineering have led to innovative surface modifications that enhance biological responses, including bone formation, particularly in compromised conditions.^15–17^ Biomedical engineering has explored new surface coatings for dental implants to promote the healing process and enhance bone formation, ultimately supporting successful implant osseointegration.^18,19^ Among these, bioactive coatings designed to stimulate osteogenesis show great potential in improving implant success rates.^20^ Notably, bioactive glass (BG) has gained attention due to its significant biological responses in both *in vitro* and *in vivo* scenarios.^21–25^ Based on this, BG has been applied to produce bioactive coatings for implant surfaces,^26–29^ representing a significant advancement in the field of implantology. Overall, these newly proposed BG-based coatings, produced by different technologies, can establish a bioactive interface that enhances cellular response, promotes osteoblast differentiation, and accelerates bone formation.^30,31^ The osteoinductive effect of BG is primarily attributed to its ionic dissolution components, which positively regulate gene expression in osteoprogenitor cells.^32^ Its biological activity is driven by the controlled release of bioactive ions (Si, Ca, P, and Na), which elevate the local pH, accelerate apatite layer formation, and enhance protein adsorption from blood plasma.^33^ This process stimulates the secretion of several growth factors, including vascular endothelial growth factor (VEGF), which plays a crucial role in angiogenesis.^32^ Additionally, BG provides a rich source of calcium and phosphate—essential components for the natural formation of bone hydroxyapatite.^34^ Furthermore, it has been shown to enhance osteoblast adhesion, enzymatic activity, and the differentiation of mesenchymal stem cells.^34^ These properties collectively position BG as a promising candidate for improving osseointegration and bone regeneration.

Among the various coating techniques explored for enhancing titanium surfaces, including the development of BG surfaces, plasma electrolytic oxidation (PEO) has emerged as a promising strategy for improving the osseointegration potential of implantable biomedical devices.^35–42^ PEO is an electrochemical oxidation process that enables the formation of complex, multiscale surface topographies while simultaneously modifying key surface properties such as roughness, surface free energy, and oxide layer composition.^42^ In the context of bone repair, *in vitro* studies have shown that the intricate topography generated by PEO coatings increases the available surface area, thereby enhancing protein adsorption and promoting better interactions between human cells and the implant surface.^43–47^ Moreover, *in vivo* evidence has shown that PEO-treated surfaces exhibit increased bone-to-implant contact, accelerated tissue repair, and a larger area of newly formed bone in femoral fractures.^35,36,38^ Importantly, PEO is an efficient technique for incorporating bioactive elements, further enhancing the beneficial effects achieved through surface modifications.^42^

Previously, Costa et al. (2020)^33^ developed and characterized a bioinspired coating that simulates the composition of the well-known 45S5-bioglass, named PEO-BG. This newly developed bioactive coating, synthesized via PEO on Ti surfaces, utilized 45S5-bioglass precursor agents (Na_2_SiO_3_-5H_2_O, C_4_H_6_O_4_Ca, NaNO_3_, and C_3_H_7_Na_2_O_6_P) in an electrolytic solution. The findings demonstrated that PEO-BG exhibited strong adhesion to the substrate, high corrosion resistance, and tribological wear, which are important properties not previously achieved with other deposition methods. In addition, PEO-BG exhibited excellent cytocompatibility with human cells, high adsorption to blood plasma proteins, and efficient formation of hydroxyapatite on its surface. These promising results have motivated us to further advance this surface modification by testing the PEO-BG coating in preclinical models under diabetes-induced conditions with the ultimate goal of translating this technology into future clinical applications. The aim is to counteract the detrimental effects of diabetes on bone repair processes.

Therefore, this study aims to assess the *in vivo* osteoinductive potential of titanium implants functionalized with a bioactive glass-based coating via plasma electrolytic oxidation (PEO-BG), focusing on their ability to promote bone regeneration under conditions of compromised healing. We hypothesize that the PEO-BG coating will enhance osseointegration by actively modulating cellular recruitment, tissue organization, and key molecular pathways involved in the process of osteogenesis. Using a preclinical model of diabetes-induced impaired bone healing, we specifically anticipate that this surface modification will improve new bone formation, support early cellular infiltration, and favorably regulate the molecular signaling pathways critical for osseointegration.

## 2 Materials and Methods

### 2.1 Experimental Design

Grade 4 commercially pure titanium implants were used in this study, supplied by DSP (DSP Biomedical, Ouro Verde II, Campo Largo-PR, Brazil). The control implants underwent surface treatment involving zirconia blasting followed by acid etching, resulting in a surface known as SLA (sandblasted, large-grit, acid-etched), as per the manufacturer’s specifications. Additionally, machined surface implants were coated with BG using PEO (PEO-BG; experimental group). First, implant surface characterizations were performed *in vitro* to check the replicability of the PEO-BG surface. Subsequently, for the preclinical study, Wistar rats were induced into a state of reduced bone mineral density through diabetes mellitus and then underwent surgery for tibial implant placement. At the time of surgery, operator blinding was not possible due to the visibly distinct topography between the control and experimental groups. However, all subsequent analyses were conducted by a blinded researcher to ensure unbiased evaluation.^48^ At 14 and 28 days postoperatively, the animals were euthanized, and the collected samples were subjected to histological, immunohistochemical, micro-CT, and histomorphometric analyses. The experimental design is depicted in Figure 1.

**Figure 1:**
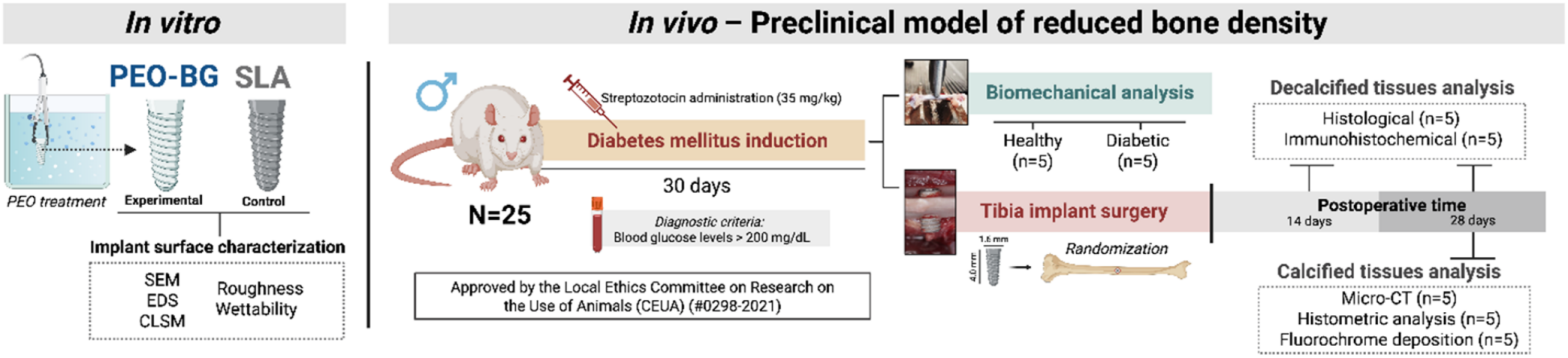
Experimental design of this study. The drawing was created with BioRender.com (License number: NG288BQJO9).

### 2.2 Synthesis of Bioactive Glass-Based Coating by Plasma Electrolytic Oxidation (PEO-BG)

For the PEO-BG coating process, the implants were treated using a DC power supply.^33^ The samples were immersed in an electrolytic solution within a stainless steel reservoir equipped with a refrigeration system, ensuring the electrolyte temperature remained stable at approximately 20 °C. The electrolytic solution composition was designed based on the 45S5 bioactive glass formulation and included the following components from Sigma-Aldrich: 0.014 M sodium metasilicate (Na_2_SiO_3_·5H_2_O; purity: 98.0%), 0.20 M calcium acetate (C_4_H_6_O_4_Ca; purity: 99.5%), 0.50 M sodium nitrate (NaNO_3_; purity: 99.5%), and 0.0010 M sodium glycerol phosphate (C_3_H_7_Na_2_O_6_P; purity: 99.0%). Additionally, 0.025 M Na2-EDTA·2H_2_O (purity: 98.0%) was used as a chelating agent in all electrolyte solutions. The PEO process was performed under the following parameters: a pulsed anodic voltage of 500 V, a frequency of 1000 Hz, and a 10% duty cycle for 420 s. After treatment, the PEO-BG-coated samples were rinsed in distilled water, air-dried, and sterilized by autoclaving for 24 h before the surgical procedure.

### 2.3 Surface Characterization

The surfaces were characterized based on their physical and chemical properties using grade II commercially pure titanium (cpTi) disks (Realum Indústria e Comércio de Metais Puros e Ligas Ltd., São Paulo, SP, Brazil) with a diameter of 10 mm and a thickness of 2 mm. The disks were polished using #320- and #400-grit SiC abrasive papers (Carbimet 2, Buehler, Lake Bluff, IL, USA) in an automatic polisher (EcoMet 300 Pro with AutoMet 250; Buehler) to standardize surface conditions. Subsequently, they were coated with surface patterns identical with those applied in the control (SLA) and experimental (PEO-BG) groups. For qualitative assessments, two samples were analyzed per group, and four samples were used for quantitative evaluations. Each analysis included readings from at least three distinct areas to ensure the reliability and validity.

#### 2.3.1 Scanning Electron Microscopy (SEM) and Energy-Dispersive Spectroscopy (EDS)

Surface morphology was examined using scanning electron microscopy (SEM), while energy-dispersive spectroscopy (EDS) was performed to assess elemental composition at the microscale (∼1 μm^3^).^35^ Elemental distribution maps were generated to visualize the spatial distribution of key chemical elements and verify the expected stoichiometric composition (atomic %) (45% Si, 24.5% Ca, 24.5% Na, 6% P; 5:2:2:1 biological ratio).

#### 2.3.2 3D Confocal Laser Scanning Microscopy (CLSM)

3D surface characterization was conducted by using confocal laser scanning microscopy (CLSM, Keyence model VK-X200 series). Both 2D and 3D images were acquired at 50 and 150× magnifications for qualitative and quantitative topographic assessments. Image processing and surface area measurements were performed using VK-Analyzer software (Keyence v 3.3.0.0).

#### 2.3.3 Surface Roughness (Profilometry)

The surface roughness profile was measured by using a profilometer. The Ra values (arithmetic mean of surface roughness) were determined using a 0.25 mm cutoff at a scanning speed of 0.05 mm/s for 12 s.

#### 2.3.4 Wettability

A goniometer (Ramé-Hart 10000; Ramé-Hart Instrument Co.) was used to evaluate the surface wettability via the sessile drop method (10 μL of DI water).

### 2.4 Experimental Design of the Animal Model

The Local Ethics Committee on Research approved this study for the Use of Animals (CEUA) (#0298–2021), and this study adhered to the ARRIVE 2.0 guidelines (Supporting File). The animals were housed in groups of four per cage in a controlled environment with a stable temperature of 22 ± 2 °C and a 12 h light/dark cycle. They had ad libitum access to solid rodent food and water. The study included 25 adult male Wistar rats (*Rattus novergicus albinus*), aged 6 months, with body weights ranging from 250 to 300 g. The sample size per group was determined using the “Sample Size for ANOVA” tool in SigmaPlot 12.0 (Exakt Graphs and Data Analysis, San Jose, California, USA), considering the primary outcome of new bone formation assessed through histometric analysis, with a mean difference of 144.30, a standard deviation of 41.13, and a statistical power of 95%, resulting in *n* = 4 tibias per group.^36^ One additional tibia was included to account for potential losses or complications, leading to *n* = 5 tibias per group for each tissue type (calcified and decalcified) and experimental period (14 and 28 days). In addition, 10 animals (5 healthy and 5 diabetic) were allocated for biomechanical analysis to confirm the reduction in bone mineral density, totaling 25 animals in the study.

### 2.5 Diabetes Mellitus Induction

To establish a preclinical model simulating reduced bone mineral density and impaired healing, diabetes mellitus was induced in the animals.^49,50^ Initially, the rats underwent a 14 to 16 h fasting period, after which blood samples were collected using the tail tip method to measure baseline glucose levels with an automatic blood glucose monitoring system (Accu-Check Performa; Roche Diagnostics Corporation, Indianapolis, IN, USA). Following this, the animals were anesthetized with an intramuscular injection of ketamine (50 mg/kg; Dopalen, Ceva Sade Animal, Paulínia, São Paulo, Brazil) and xylazine hydrochloride (5 mg/kg; Anasedan, Ceva Sade Animal, Paulínia, São Paulo, Brazil). Under anesthesia, they received an intravenous injection of streptozotocin (Sigma-Aldrich, St Louis, MO, USA) dissolved in a citrate buffer solution at a concentration of 35 mg/kg, administered via the penile vein.^49,51–53^ Six days after diabetes induction, blood samples were collected again to determine the blood glucose levels, and animals with values exceeding 200 mg/dL were included in the study. This threshold aligns with the diagnostic criteria for diabetes in humans based on random blood glucose levels in symptomatic individuals.^54^ Over the subsequent 30 days, weekly blood glucose measurements were performed to monitor the progression of diabetes. After this period, the animals underwent surgery to install titanium implants in the tibia, allowing for the evaluation of bone healing under conditions of compromised bone metabolism.

### 2.6 Analysis of Bone Mineral Density (Biomechanical Analysis)

A biomechanical analysis confirmed the reduced bone mineral density as a result of diabetes. For this purpose, 10 animals were separated from the original sample and divided into two groups: (1) the diabetic group (*n* = 5), which underwent the diabetes induction via streptozotocin, and (2) the healthy group (*n* = 5), which underwent a simulation of diabetes induction but did not receive the streptozotocin solution. The three-point bending test (MZ-500S; Maruto Instrument) was conducted. The tibias were positioned on a platform supported by two points—an anterior and a posterior—ensuring stabilization. A vertical force was then applied to the central region of the tibia at a rate of 5 mm/min. The resulting deformation and load curves were plotted, allowing for the assessment of stiffness and the maximum force applied to the tibias.^35^

### 2.7 Tibial Implant Surgery and Placement

Thirty days after the induction of experimental diabetes, the animals were fasted for 12 h before surgery. Subsequently, they were anesthetized with a combination of 70 mg/kg of ketamine and 5 mg/kg of xylazine administered intramuscularly. Local anesthesia was then applied using mepivacaine hydrochloride (0.3 mL/kg, Mepiadre 2% 1:100,000, Nova DFL, Rio de Janeiro, Brazil). The surgeries were performed by a single operator (S.B.), and the surgical procedure for implant installation followed the methodology established in previous studies by our group.^36,55,56^ Titanium implants measuring 1.6 mm in diameter and 4 mm in height were installed, with one implant per tibia in each animal chosen randomly. After implantation, the tissues were repositioned and sutured in layers. Each animal received both implants related to the study (PEO-BG or SLA), and the selection of the tibia (right or left) for surgery was performed by a nonoperating participant (L.P.F.), who used envelopes containing the tibia sides. Accordingly, surgical procedures were performed on 30 tibiae to place implants.

At 14 and 28 days postoperatively, the animals were euthanized through transcardiac perfusion with physiological saline solution supplemented with 0.1% heparin, followed by 4% formaldehyde fixative solution (Sigma-Aldrich) in phosphate-buffered saline (PBS, Sigma-Aldrich), 0.1 M, pH 7.4, at 4 °C.^57^ After euthanasia, the tibiae were collected and processed for further analyses. The analyses were performed on two types of tissue: decalcified tissues, which were evaluated at both 14 and 28 days, and calcified tissues, which were assessed only at 28 days.

### 2.8 Analysis of Decalcified Tissues

#### 2.8.1 Histological Analysis

At 14 and 28 days postoperatively, five tibias from each group were subjected to EDTA (10%) decalcification for 90 days. After decalcification, the samples were dehydrated, diaphanized, and embedded in paraffin. Subsequently, 5 μm thick histological sections were prepared.^58–61^ The implants were carefully removed from the tibias before embedding. Following microtomy, the slides were stained with hematoxylin and eosin (HE) for qualitative analysis, as described below.

##### 2.8.1.1 Qualitative Evaluation of Bone Tissue

To observe the maturation pattern and characteristics of bone tissue in the different groups, photomicrographs were taken with a 40× objective. All images were subsequently analyzed by a blinded and calibrated examiner.^62^

##### 2.8.1.2 Inflammatory Profile Assessment

To assess the inflammatory profile of each group, with a particular focus on mononuclear inflammatory cells and blood vessels, three photomicrographs were captured from each sample using a 100× objective in different regions of the central spiral and bone marrow area. ImageJ software “Grid” and “Cell Counter-Notice” tools were then used to locate and count inflammatory cells and blood vessels.

#### 2.8.2 Immunohistochemical Analysis

The odd-numbered slides from the microtomy were subjected to immunohistochemical reactions using the following primary antibodies from Santa Cruz Biotechnology (Dallas, TX, USA): anti-BMP-2 (BMP-2; sc-137087), osteoprotegerin (OPG; sc-390518), receptor activator of nuclear factor kappa B ligand (RANKL; sc-377079), and osteocalcin (OC; sc-365797). The goal was to analyze cellular responses related to induction and formation (BMP-2), remodeling (OPG and RANKL), and bone mineralization (OC). The immunohistochemical reactions were processed according to established methodologies.^55,63,64^ For each antibody, protein expression was quantified by counting the marked cells.^65,66^ The images were captured using a 100× lens in three distinct areas of the most central medullary bone tissue. The marked cells were counted using the ImageJ “Grid” “Crosses” tool, with a total of 130 “crosses” per image.^67^

### 2.9 Analysis of Calcified Tissues

#### 2.9.1 Microcomputed Tomography (Micro-CT)

At 28 days postoperatively, some of the animals were euthanized, and five tibias from each group were scanned using a SkyScan microtomography scanner (SkyScan 1176 Bruker Micro-CT, Aatselaar, Belgium). After reconstruction in NRecon (SkyScan, 2011; Version 1.6.6.0) and three-dimensional repositioning in Data Viewer (SkyScan, Version 1.4.4 64-bit), the images were transferred to CTAn CTAnalyzer (2003–11 SkyScan, 2012 Bruker Micro-CT Version 1.12.4.0). In CTAn, the implant was removed through a sequential task list, and the region of interest (ROI) was determined. This region comprises the entire medullary bone region formed between the implant coils. Corticalized bone tissue was disregarded for all samples, as this does not represent newly formed bone tissue. The quantitative patterns of bone tissue were expressed in terms of bone volume (BV) and bone volume-to-total volume ratio (BV/TV, representing the percentage of bone volume). Meanwhile, the quality of the newly formed bone tissue was assessed using trabecular thickness (Tb.Th), trabecular separation (Tb.SP), and the number of trabeculae (Tb.N).^62^

#### 2.9.2 Determination of Bone Turnover through Fluorochrome Deposition

Following microtomography, the tibiae were processed into calcified slides using the EXAKT system (Cutting System, Apparatebau, Gmbh, Hamburg, Germany). After dehydration and inclusion in Techno Vit photopolymerizable resin (Germany, Heraeus Kulzer GmbH, Division Technik, Philipp-Reis-Str. 8/13, D-61273 Wehrheim), the samples were cut and polished to obtain a section approximately 60 μm in thickness. The slides were then analyzed using a light microscope (Model BX53; Olympus) with a fluorescence system and an attached color camera (XC50, Olympus) to assess the deposition of the fluorochromes calcein and alizarin, which were administered on days 14 and 21 postsurgery, respectively. The images were captured using color filters 3 and 4 to obtain red and green individual images, which were later combined using the “CellSens” program. The deposition area of each fluorochrome was quantified with images taken at 10× magnification, ensuring no color overlap. A triangular region located between the two most central threads of the implant situated within the medullary bone tissue was selected. This area was measured and designated as the total area (100%). Subsequently, each deposition was chosen using the “Free hands” tool in ImageJ, and the area was quantified in μm^2^ and converted into a percentage (%).

#### 2.9.3 Histometric Analysis

After being processed, the same sections were washed in deionized water and stained with Alizarin red and Stevens blue to label the newly formed bone tissue. The photomicrographs were then opened using ImageJ software for further analysis. For the analysis of the newly formed bone area (NBA), a triangular region located between the two most central threads of the implant, situated within the medullary bone tissue, was measured and designated as the total area (100%). Subsequently, using the “freehand” tool in ImageJ, the area of newly formed bone was quantified in μm^2^ and converted into a percentage (%) relative to the total area. Similarly, the triangular region between the two most central threads had its total extent determined and considered as the total length (100%). Thereafter, the linear extent of bone–implant contact (BIC) was measured using the “straight” tool in ImageJ, quantified in μm^2^, and the value was converted into a percentage (%).

### 2.10 Statistical Analysis

All quantitative response variables in groups (PEO-BG and SLA) were initially subjected to the Shapiro–Wilk normality test to determine whether the data followed a normal distribution. For the parameters of wettability, roughness, and surface area, Student’s *t* test was applied. Similarly, the *t* test was used for biomechanical analysis, micro-CT, and histometric comparisons to assess the differences between the groups. A two-way ANOVA was performed to evaluate the interaction between the different factors (groups and time) for histology, immunohistochemistry, and fluorochrome deposition area. All statistical analyses were conducted using GraphPad Prism version 8.0.

## 3 Results

### 3.1 Enhanced Surface Roughness and Hydrophilicity of PEO-BG Coating Compared to SLA

The PEO-BG coating was produced using the same method and parameters established in our previous study, which confirmed the presence of BG on the Ti surface.^33^ The SEM micrographs revealed that the SLA surface (control) exhibited irregularities distributed in a nonhomogeneous pattern, with the majority of these irregularities being small cavities typical of the surface treatment subtraction processes, as expected for this type of surface (Figure 2a). In contrast, the PEO-BG surface displayed numerous aggregates with irregular contours, which were uniformly distributed across the surface, contributing to an increased surface roughness (Figure 2a). The EDS analysis showed a higher percentage of oxygen relative to Ti on the PEO-BG surface, along with the successful incorporation of ions from the electrolytic solution that mimics the composition of bioglass, including Si, Ca, P, and Na, confirming the BG composition (Figure 2b). CLSM images revealed distinct surface characteristics of the two groups. The PEO-BG group exhibited pronounced roughness with noticeable height variations between peaks and valleys, whereas the SLA group had a flatter profile (Figure 2c). It was confirmed by the superior roughness of the PEO-BG coating (*p* < 0.001) (Figure 2d). PEO-BG also displayed greater hydrophilicity, as indicated by a significantly lower water contact angle (31.02°) compared to the SLA surface (78.20°) (*p* = 0.029) (Figure 2e).

**Figure 2:**
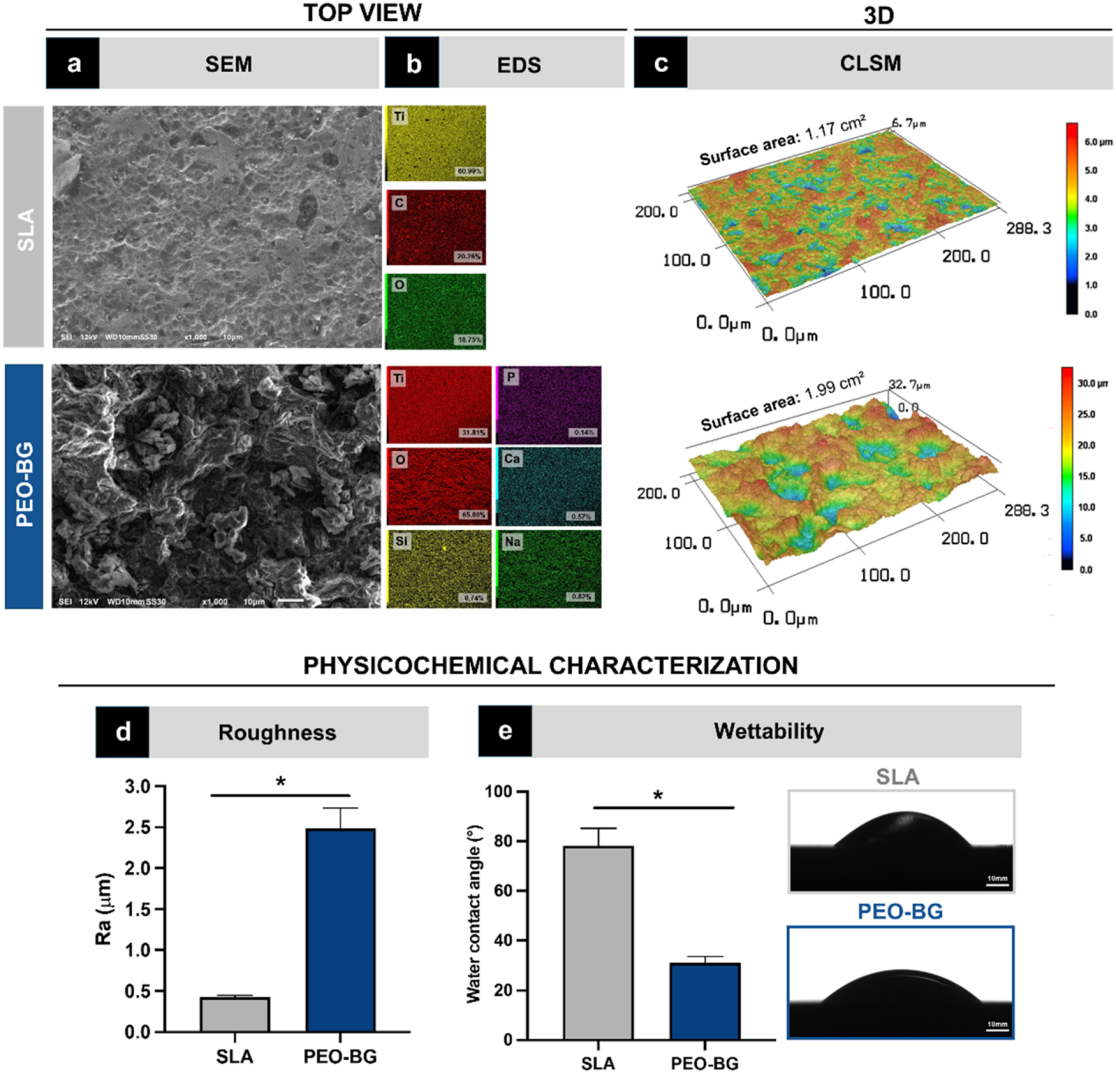
Surface characteristics of PEO-BG and SLA groups. (a) Scanning electron microscopy (SEM) of the surfaces; (b) energy-dispersive spectroscopy (EDS) of the surfaces showing the spatial distribution of key chemical elements and confirming the expected stoichiometric composition of a bioactive bioglass (atomic %); and (c) 3D confocal laser scanning microscopy (CLSM) characterization of the surfaces. Both 2D and 3D images were acquired at 50× and 150× magnifications for qualitative and quantitative topographic assessments. (d) Surface roughness average. (e) Wettability. Statistically significant differences between groups are marked with an asterisk (*) (*p* < 0.05).

### 3.2 Proof of Concept: Bone Mineral Density Is Reduced in the Diabetic Rat

Biomechanical analysis revealed that diabetes-induced rats exhibited significantly lower bone stiffness (121.45 kN/m  ±  15.79) compared to healthy controls (168.13 kN/m  ±  9.66; *p* = 0.002) (Figure 3a). Similarly, maximum force measurements were also significantly reduced in diabetic animals (79.73 N ±  8.61) relative to healthy counterparts (101.79 N ±  5.36; *p* < 0.002) (Figure 3b), supporting the observation of reduced bone mineral density.

**Figure 3:**
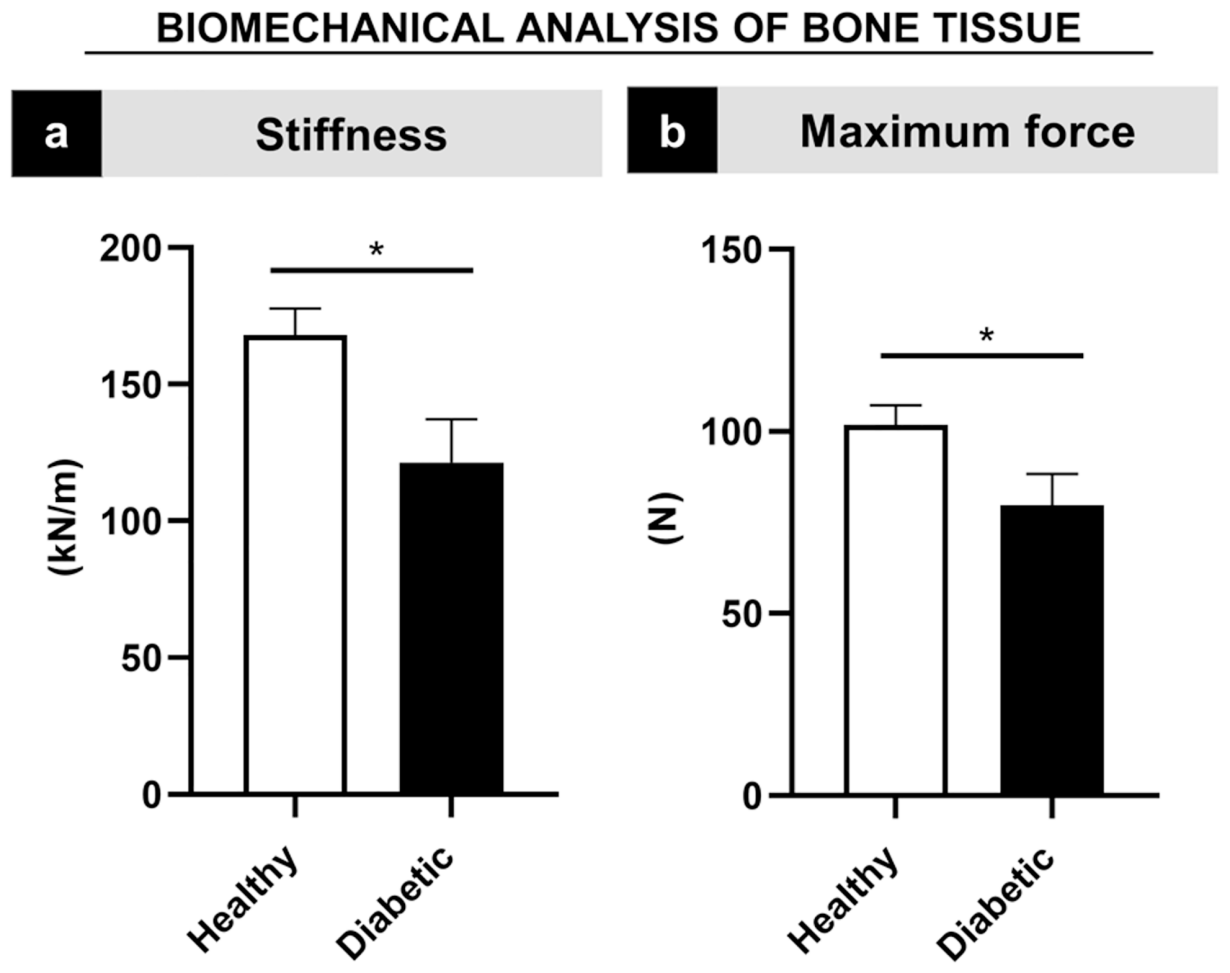
Biomechanical analysis of bone tissue characterization in diabetic animals compared with healthy animals. (a) Stiffness. (b) Maximum force. Statistically significant differences between groups are marked with an asterisk (*) (*p* < 0.05).

### 3.3 PEO-BG Accelerates Bone Regeneration by Promoting Increased Bone Formation, Reducing Inflammation, and Enhancing the Expression of Key Proteins Involved in the Repair Process

Given the chemical composition of the PEO-BG coating and its ability to promote favorable biological responses, we investigated whether this surface modification could enhance bone formation in diabetes-induced rats with impaired bone density. The histological data revealed a more mature repair pattern in the PEO-BG group at both postoperative time points, evidenced by greater bone formation, improved cellular organization, and a lower presence of connective tissue cells compared to that of the control group (Figure 4a). Regarding the inflammatory profile, which focused on mononuclear inflammatory cell and blood vessel counts, both groups exhibited a similar inflammatory pattern at 14 days (*p* > 0.05) (Figure 4b,c). However, by 28 days, the SLA group presented a higher inflammatory cell count (*p* = 0.001) and a lower number of blood vessels (*p* < 0.001) (Figure 4b,c), which may suggest a more persistent inflammation in the control group. In the intragroup analysis, comparing 14 and 28 days, the SLA group exhibited an increased inflammatory cell count, while the PEO-BG group showed a higher blood vessel count (Figure 4c).

**Figure 4:**
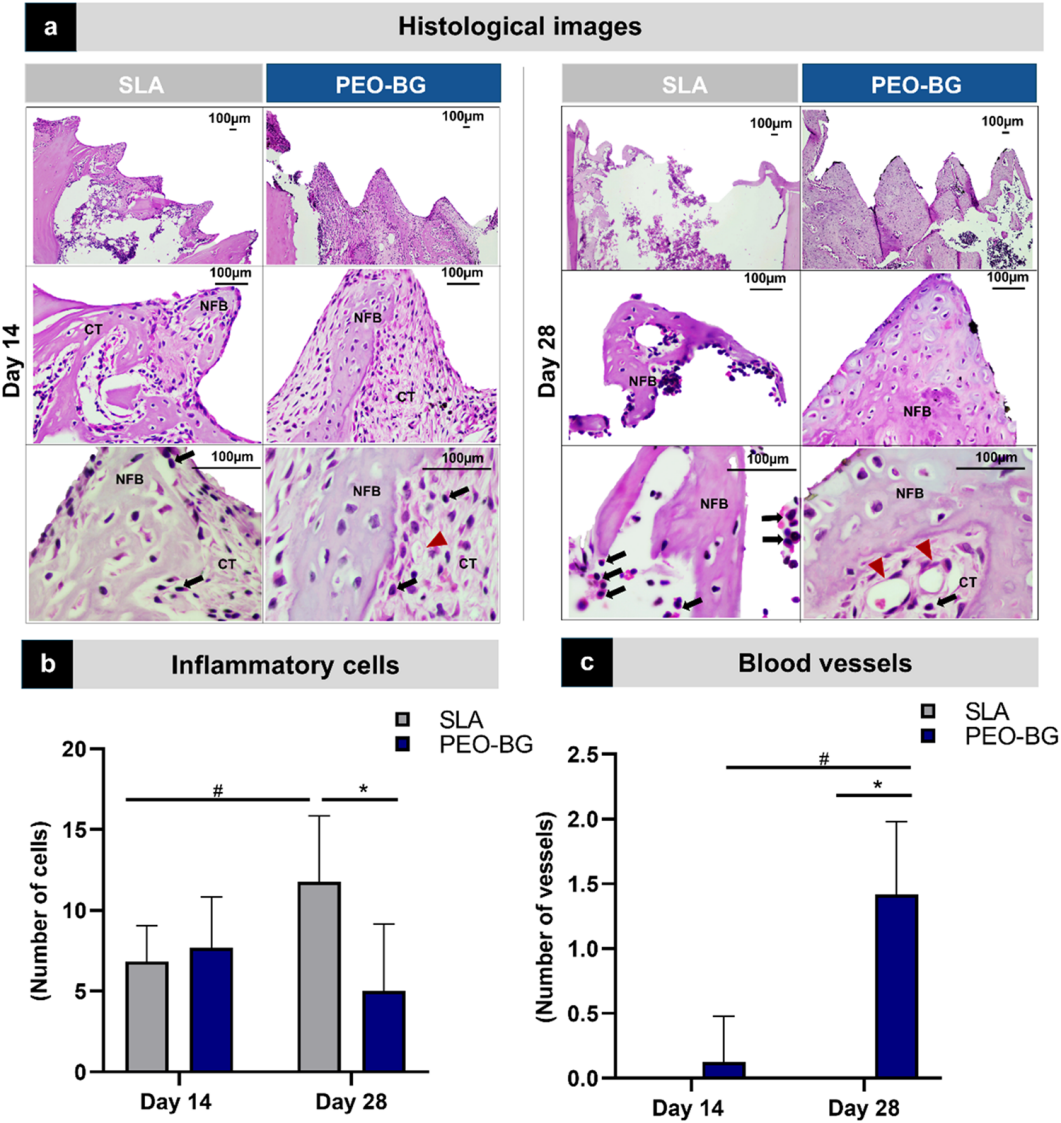
Histological analysis. (a) Representative photomicrographs at 10×, 40×, and 100× magnifications taken at 14 and 28 days postoperatively, comparing SLA (control) and PEO-BG groups. (b) Inflammatory cell counts for SLA (gray) and PEO-BG (blue) groups. (c) Graphical representation of blood vessel count for the SLA (gray) and PEO-BG (blue) groups. Black arrows indicate the cell pattern considered in the count (lymphocytes). Red arrows indicate blood vessels. NFB–Newly formed bone; CT–Connective tissue. Statistically significant differences between groups are marked with an asterisk (*), while intragroup differences are indicated by a hash symbol (#) (*p* < 0.05).

In the immunohistochemical analysis, both implant surfaces (SLA and PEO-BG) exhibited expression of all assessed proteins—BMP-2, OPG, RANKL, and OCN—even if the expression was slight in some samples (Figure 5a). BMP-2 immunostaining was more pronounced in the PEO-BG group at both postoperative time points, with significantly higher expression at 14 days (*p* = 0.048; Figure 5b). Similarly, OPG and RANKL expressions were significantly higher in the PEO-BG at both time points (*p* < 0.05) (Figure 5c,d). In contrast, the level of OCN expression was higher in the SLA group at 14 days (*p* = 0.017), but by 28 days, no significant difference was observed between the groups (Figure 5e). Since these proteins are essential biomarkers indicative of bone formation and healing, reflecting osteoinductive potential and cell differentiation activity, the expression profile suggests enhanced activity in the PEO-BG group, particularly at early time points.

**Figure 5:**
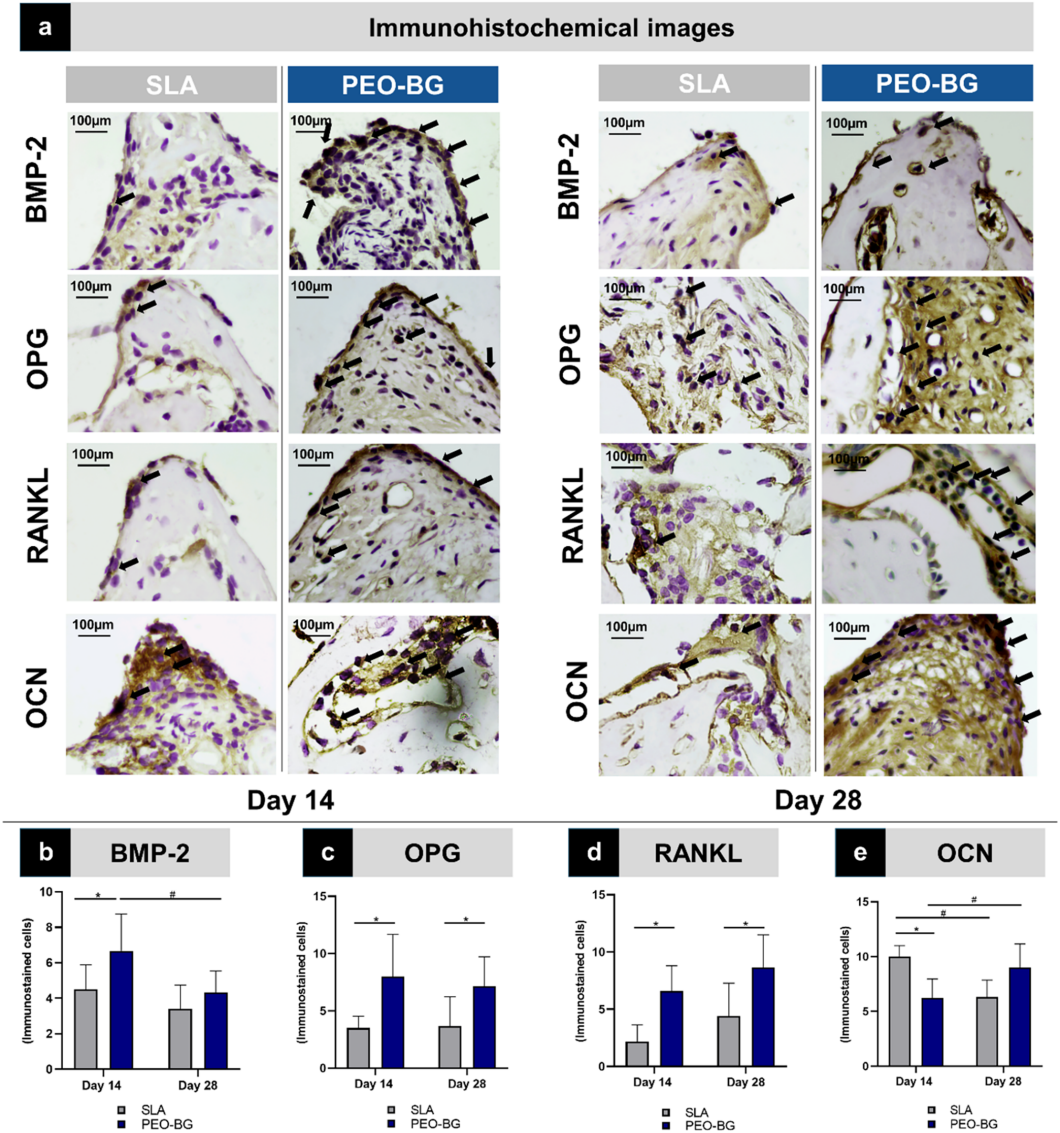
Immunohistochemical analysis. (a) Photomicrographs of the coil regions with immunostaining for the respective proteins at 14 days postoperatively and 28 days postoperatively for SLA (control) and PEO-BG groups. (b) BMP-2 immunostaining quantification. (c) OPG immunostaining quantification. (d) RANKL immunostaining quantification. (e) OCN immunostaining quantification. Statistically significant differences between groups are marked with an asterisk (*), while intragroup differences are indicated by a hash symbol (#) (*p* < 0.05).

### 3.4 PEO-BG Improved Bone Formation and Quality under Diabetic Conditions by Enhancing Bone Volume, Accelerating Bone Turnover, and Promoting Implant Integration

Computed microtomography revealed distinct patterns of bone formation among the groups. A representative 3D image illustrates that the PEO-BG group developed more robust coils with a homogeneous distribution around the implant. In contrast, the SLA (control) group exhibited thinner and irregularly distributed coils (Figure 6a), showing the significant effect of PEO-BG in improving bone formation, even under conditions of impaired bone density. This outcome was confirmed by quantitative analysis, which showed that bone volume (BV) and the percentage of bone volume (BV/TV) were significantly higher in the PEO-BG group (*p* < 0.001) (Figure 6b). In terms of bone quality parameters, the trabecular thickness (Tb.Th) was comparable between the groups. However, trabecular separation (Tb.Sp) was greater in group SLA (*p* = 0.029), while the number of trabeculae (Tb.N) was higher in the PEO-BG (*p* = 0.029) (Figure 6c).

**Figure 6:**
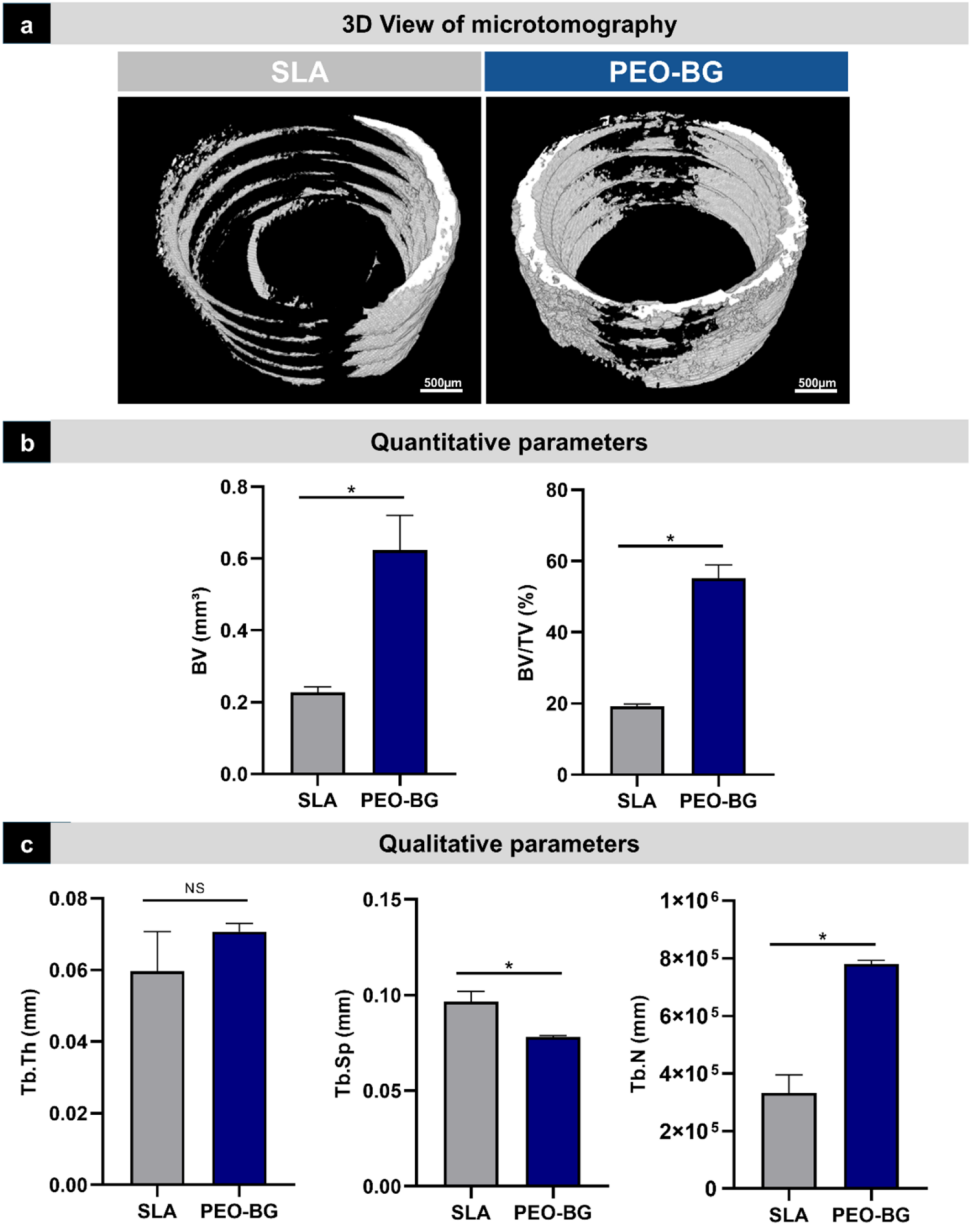
Microtomographic analysis. (a) Three-dimensional representation of the bone area around the implant. (b) Quantitative microtomographic data: bone volume (BV) and percentage of bone volume (BV/TV). (c) Estimate parameters for trabecular thickness (Tb.Th), trabecular separation (Tb.Sp), and trabecular number (Tb.N). Statistical differences are indicated by the asterisk symbol (*) (*p* < 0.05). NS – Not significant.

Fluorochrome labeling, used to assess bone turnover, revealed that the PEO-BG group presented larger areas of alizarin labeling, indicative of more recently deposited bone (Figure 7a). This is particularly evident in the 10× magnification images (Figure 7a), where intense fluorescent regions corresponded to the fluorochrome incorporation. In contrast, the SLA group showed minimal alizarin labeling and greater calcein deposition, suggesting delayed bone formation. Quantitative analysis confirmed significantly higher incorporation of both calcein (green) and alizarin (red) in the PEO-BG group compared to SLA (*p* < 0.001) (Figure 7b). Additionally, the intragroup comparison for calcein and alizarin deposition also showed a statistically significant difference for both PEO-BG and SLA. For SLA, there was a higher deposition of calcein (older bone). Meanwhile, PEO-BG was characterized by greater alizarin deposition (newly formed bone).

**Figure 7:**
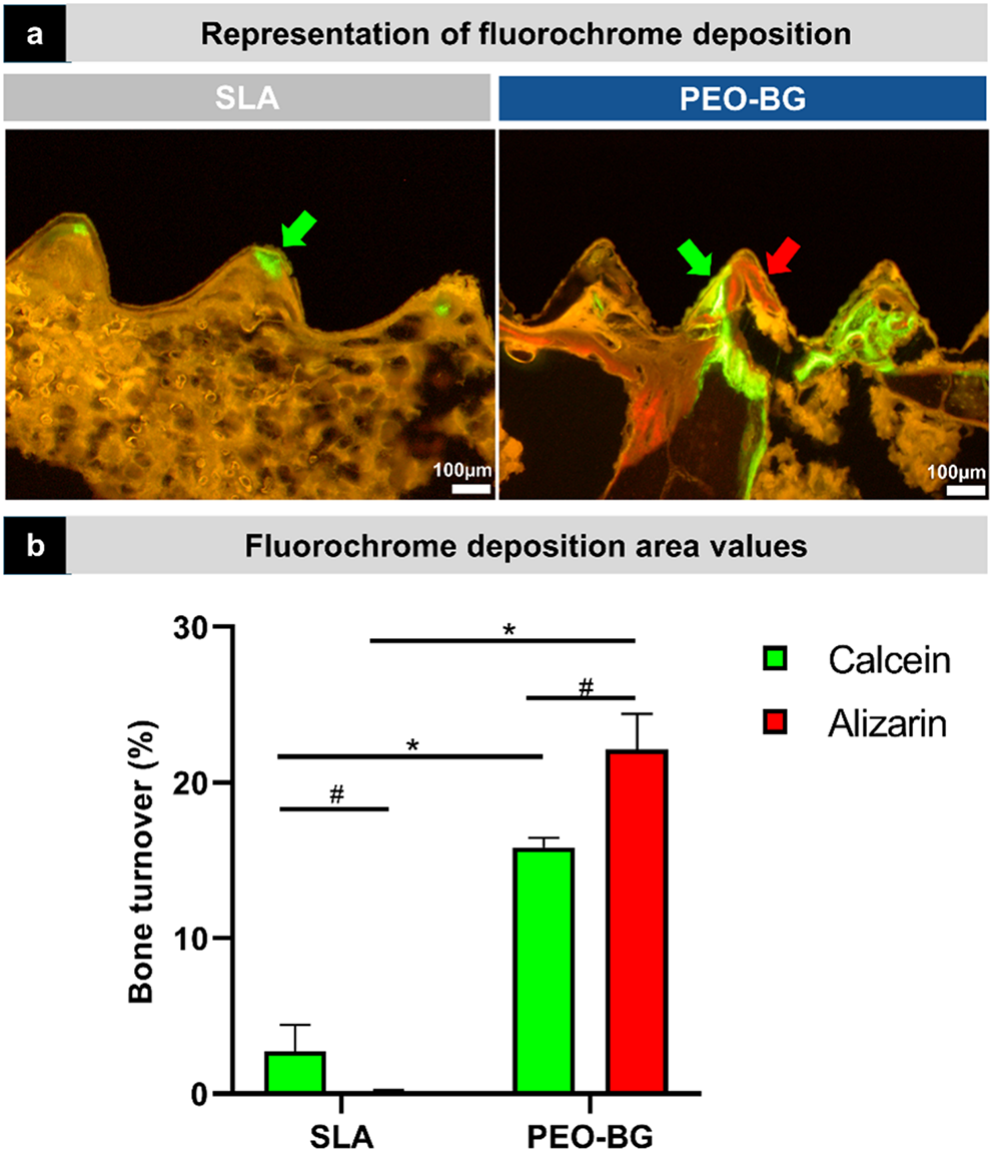
Fluorochrome deposition area analysis for calcein (green) and alizarin (red). (a) Representative images of the SLA and PEO-BG groups at 10× magnification. The green fluorescence (green arrow) corresponds to calcein deposition, which represents the older bone tissue in both groups. The red, present only in the PEO-BG group (red arrow), represents the newly formed bone tissue. (b) Fluorochrome deposition area values. Statistical differences between groups are indicated by the asterisk symbol (*), and intragroup differences by the hash symbol (#) (*p* < 0.05).

Histometric analysis and imaging demonstrated that the PEO-BG surface promoted more effective bone infill between implant threads, indicating accelerated and enhanced bone formation (Figure 8a). In contrast, the SLA group exhibited smaller areas of newly formed bone, irregularly distributed within the central region of the defect (Figure 8a). Quantitative analysis revealed significantly higher values for the neoformed bone area (NBA: SLA = 4.77 × 10^6^ μm^2^  ±  1.22 × 10^6^; PEO-BG = 8.52 × 10^7^ μm^2^  ±  9.40 × 10^6^) and bone-to-implant contact (BIC: SLA = 1.42 × 10^5^ μm  ±  3.57 × 10^4^; PEO-BG = 6.35 × 10^5^ μm  ±  1.27 × 10^5^) in the PEO-BG group (*p* < 0.005) (Figure 8b). (Figure 8b). These results confirm the ability of the PEO-BG coating to stimulate and accelerate peri-implant bone regeneration, even under conditions of impaired bone density associated with diabetes.

**Figure 8:**
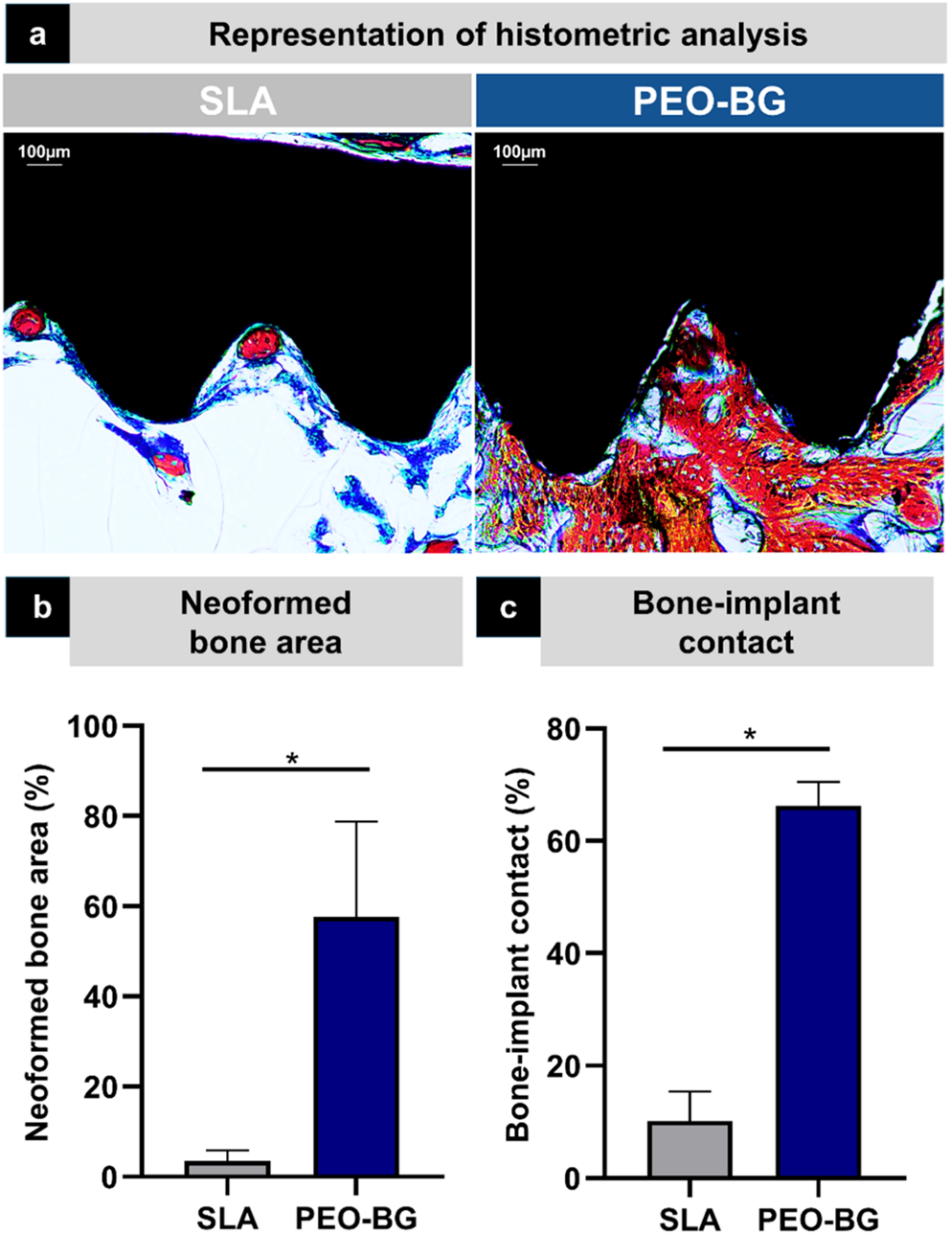
Histometric analysis. (a) Photomicrographs of calcified slides demonstrating greater bone formation in the PEO-BG. (b) NBA and (c) BIC values are both significantly higher in the PEO-BG group. Statistically significant intergroup differences are indicated by an asterisk (*) (*p* < 0.05).

## 4 Discussion

The development of bioactive coatings for implantable devices represents a significant advancement in precision medicine, particularly in improving osseointegration for challenging clinical conditions, such as diabetes. Bone tissue growth is a dynamic process, and the use of responsive surfaces that meet the requirements to stimulate bone regeneration represents a promising strategy in biomedical engineering.^68^ Tissue engineering has explored the potential of BG due to its high osteogenic capacity and ability to promote bone regeneration.^69^ In fact, *in vitro* and *in vivo* evidence have shown that BG enhances the expression of bone morphogenic proteins,^70^ exhibits strong osteoconductive properties,^71^ promotes bone cell proliferation,^72^ and ultimately accelerates osseointegration.^73^ Moreover, BG has been explored as a potent agent to enhance bone regeneration in diabetic microenvironments when utilized as a hydrogel scaffold.^74^ In addition to their bioactive properties, BG coatings for dental implant applications must also exhibit enhanced mechanical strength to withstand the challenging conditions of the oral environment.

Importantly, we have previously shown that the BG coating used here exhibits proper biocompatibility *in vitro*, enhanced mechanical and tribological properties, and higher corrosion resistance, which enables the control of overgrowth of pathogenic species related to implant-related infections,^33^ the primary reason for implant treatment failures.^75^ The PEO-BG coating resulted in a significant increase in protein adsorption (∼2-fold, *p* < 0.05), making it a suitable candidate for supporting early-stage osseointegration. Moreover, the PEO-BG coating demonstrated cytocompatibility with fibroblast cells and promoted a distinct cellular organization.^33^ The rough surface topography induced fibroblasts to arrange in a spatial pattern, resulting in more homogeneous coverage, with cells exhibiting a stellate morphology characteristic of young, proliferating cells.^33^ These biological responses can be attributed to the surface roughness and the chemical composition of PEO-BG, which resembles the well-known bioactive glass 45S5, obtained through plasma electrolytic oxidation. Here, we moved forward to the next step, testing the effective BG coating in a preclinical model and focusing on a profile that could benefit most from this technology: diabetes patients, who represent a clinical challenge for bone formation and healing. The findings present a novel strategy aimed at enhancing the biological response at the implant–bone interface, potentially mitigating complications associated with poor bone quality, such as in diabetes and other pathological conditions that compromise bone quality. In these populations, impaired vascularization and reduced osteoblastic activity can hinder optimal implant integration, making conventional implant surfaces less effective.

Diabetes and its consequently high glucose levels, when poorly controlled, have a direct impact on bone quality and healing processes, which can compromise the osseointegration of implant devices. High glucose levels in diabetes may exert toxic effects on the differentiation of bone marrow mesenchymal cells,^76^ increase the expression of sclerostin, a negative regulator of bone formation,^76^ enhance the expression of inflammatory cytokines, and promote elevated levels of reactive oxygen species (ROS).^77^ In fact, streptozotocin-induced diabetes in rats has been shown to result in increased ROS expression, reduced proliferation of bone marrow-derived mesenchymal stem cells, and, consequently, reduced bone–implant contact and implant survival rates.^78^ Here, using a streptozotocin-induced diabetes animal model, we demonstrated an innovative and effective PEO-BG coating that counteracts these diabetes-related consequences, promotes bone formation, and controls inflammation, thereby enhancing implant osseointegration. By adopting a biomimetic approach, our coating fostered favorable cellular interactions, boosting osteoblast activity and modulating the inflammatory response. This is critical for preventing early implant failure and enhancing the overall clinical outcomes. The results of this preclinical study clearly demonstrated the osteoconductive and osteoinductive properties of the developed PEO-BG coating, highlighting its fundamental role in bone repair.

Most commercially available implant surfaces are considered osteoconductive because, in addition to being biocompatible, they possess surface energy that enables good wettability, promoting protein adhesion and osteoprogenitor cell attachment.^79^ On the other hand, osteoinduction represents a more complex process. It is currently found in a few materials related to bone repair, such as autogenous bone and human bone morphogenetic protein (BMP).^80^ Osteoconduction refers to the ability of a material or coating to facilitate interaction with undifferentiated mesenchymal cells, serving as a scaffold for their differentiation into osteogenic lineage cells. In contrast, osteoinduction refers to the ability of a material or coating to actively induce undifferentiated mesenchymal cells into osteoblasts, thereby directly stimulating bone repair.^81^ BG has been extensively studied for bone regeneration and tissue engineering due to its osteoinductive activity, particularly related to silica content.^34,82,83^ The degradation of silica produces ionic products that interact with adjacent tissues, stimulating cell attraction and differentiation.^84^ In this context, EDS analysis is crucial in demonstrating the effective incorporation of bioglass components into the coating. This is especially important for silica as its osteoinductive properties represent a key distinguishing feature of the PEO-BG coating, setting it apart from other commercially available coatings.

An essential mechanism of BG materials is their ability to induce hydroxyapatite (HAp) formation. Due to ionic exchange between the coating and surrounding fluids, Ca^2+^ and PO_4_^3–^ ions—the main components of HAp—diffuse from the PEO-BG coating and, together with similar ions present in the fluid, lead to the formation of HAp that is chemically and structurally similar to the mineral apatite phase found in bone tissue.^33^ Due to its structural and functional similarity to the inorganic component of natural bone, HAp plays a crucial role in promoting bone cell proliferation and osseointegration.^85^ Moreover, HAp has shown important immunomodulatory properties, creating a favorable osteoimmune microenvironment.^85^ Aligned with the chemical composition of BG, the topography generated by the PEO provides excellent mechanical and biological outcomes to the implant surface, also promoting bone formation. PEO surfaces show favorable topographic changes associated with the enriched composition of the oxide layer, increased surface energy, and improved interaction with undifferentiated mesenchymal cells.^13,53^ In preclinical experimental models, PEO has demonstrated that the topographic changes induced by the anodization technique enhance bone repair, particularly in bones with a reduced mineral density. This enhancement in repair was primarily related to increased contact area, improved wettability, and the presence of calcium and phosphate—favorable conditions for protein and osteogenic cell interactions.^35,36,38^ Therefore, the PEO-BG coating combined chemical and topographical modifications that promoted bone formation and healing, even under conditions of impaired bone density, representing a promising strategy for future clinical translation and testing.

Here, using a PEO-BG surface, this study has demonstrated the reproducibility of the surface, its topographic advantages, and its reparative effects in a preclinical model characterized by bone mineralization deficiency, thereby emphasizing the osteoconductive properties of the developed PEO-BG coating. A key finding in the PEO-BG group was the presence of blood vessels near the implant threads. Some vessels were seen at 14 days, with an increase observed after 28 days. In contrast, the SLA group showed no vessels throughout the experimental period, reflecting a much slower rate of bone formation around the implant surface. This improved vascularization is likely linked to the proangiogenic effects of the bioactive glass-inspired coating. Bioactive glass regulates gene secretion by stimulating growth factors, such as vascular endothelial growth factor (VEGF) and basic fibroblast growth factor (bFGF).^32,86^ The histological analysis showed that the peri-implant region in the PEO-BG group was characterized by organized tissue, fewer inflammatory cells, and a higher presence of osteoblasts and vessels. This suggests more mature bone formation in comparison to the SLA group. In the SLA group, while bone neoformation occurred, it was less pronounced with smaller spirals and disorganized tissue. The inflammatory profile further demonstrated that the SLA group had greater inflammatory infiltrates, which are known to hinder bone repair.^87,88^ Histometric analysis revealed one of the primary outcomes of this study: the peri-implant region in the PEO-BG group had a significantly larger area of newly formed bone. Although the samples were evaluated only up to 28 days postoperatively, the PEO-BG group showed an advanced attempt to fill the spaces between the implant coils with more structured bone tissue and a greater linear extent of bone-to-implant contact. These results suggest that osseointegration was notably enhanced in the PEO-BG group, even within a relatively short postoperative period.

The micro-CT analysis reinforced the histometric findings, revealing significantly higher bone volume and bone volume percentage in the PEO-BG. This was particularly evident in the 3D representation, which displayed more robust coils and a more homogeneous bone distribution around the implant after 28 days. Furthermore, in terms of neoformed bone quality, the PEO-BG group presented a greater number of bone trabeculae, whereas the SLA group showed more widely separated trabeculae. PEO has demonstrated abilities to accelerate and optimize bone repair, yielding outcomes comparable to those observed in healthy animals, with no detectable alterations in bone mineral density.^35–37^ The immunostaining results for key bone-related proteins further supported the histological and histometric findings. BMP-2, a marker of early bone formation, exhibited significantly higher expression at 14 days in the PEO-BG group, correlating with increased osteoblast recruitment and higher bone neoformation during this period. Similarly, RANKL and OPG, proteins involved in regulating osteoclast activity and bone resorption and deposition, were also significantly more expressed in the PEO-BG group than in the SLA. This demonstrates a higher rate of bone turnover, which is a crucial factor for tissue renewal and bone matrix deposition. In turn, OCN, a marker of bone mineral matrix deposition, showed significantly higher expression in the SLA group at 14 days. However, by 28 days, this trend reversed with PEO-BG showing slightly higher levels. Considering the OCN results alongside other findings, it is plausible that the SLA group displayed superior immunostaining at 14 days due to the delayed mineralization process. Meanwhile, PEO-BG had already progressed to a more advanced phase of bone formation, demonstrating the earlier maturation of the newly formed bone tissue. The deposition pattern of the fluorochromes calcein and alizarin also corroborates the finding of accelerated bone remodeling. Besides having less newly formed bone, the SLA group showed insignificant values of newly formed bone (as indicated by alizarin) compared with the PEO-BG group. Meanwhile, PEO-BG demonstrated satisfactory values of older, newly formed bone deposition, but still with higher values of recently formed bone, indicating that significant bone remodeling had occurred. Greater alizarin depositions were also observed by Momesso et al. (2020) on surfaces modified by plasma electrolytic oxidation.^38^

Given the enhanced bone repair observed in the peri-implant region with the PEO-BG surface compared to that with the SLA surface, it is reasonable to anticipate that this coating technology substantially improves osseointegration, particularly in challenging clinical scenarios where bone healing is compromised. It is important to recognize that patients with decompensated systemic conditions often experience implant or orthopedic failures, necessitating surgical reinterventions. These complications increase morbidity and contribute to higher treatment costs and prolonged recovery periods.^89,90^ The main limitation of this study lies in the extrapolation of the animal model results to clinical applications. While the tibia is a widely used model for bone healing studies, it may not fully replicate the conditions of the alveolar bone in patients with compromised bone mineral density.

Furthermore, when considering the clinical application of the coating for biomedical implants, we believe that future studies should evaluate the coating’s degradation during implant insertion and removal to validate *in vitro* results under clinically relevant conditions. It is expected that the implant placement surgery will not alter the greater wear resistance, higher mechanical strength, and corrosion resistance previously demonstrated by PEO-BG.^33^ It is important to emphasize that the mechanical resistance mentioned here refers to the physical properties of the surface itself rather than to the bulk material used in the fabrication of the titanium disks and implants. Although grade 2 titanium disks and grade 4 titanium implants were used due to availability and manufacturing convenience, the minimal compositional differences between these grades, as specified by ASTM B348, do not influence the outcomes of this study, as only properties related to surface topography and the corresponding biological responses were evaluated. Given the mechanical and chemical stability of PEO-based coatings, our findings suggest that standard autoclave sterilization (121–134 °C for 30 min) does not compromise the integrity or performance of the surface.^91,92^ Nonetheless, the standardization of sterilization protocols should be considered in future studies to ensure coating stability in large-scale manufacturing and clinical applications.

This approach aligns with the principles of precision medicine, offering a patient-specific solution that addresses factors such as age-related bone loss and systemic conditions that affect bone metabolism, including diabetes. Our findings highlight PEO-BG as a surface that is easily reproducible and yields highly favorable results for repair. Future studies investigating different implant loading times in bone models with low mineral density—particularly in the posterior regions of the maxilla or mandible—could provide valuable insights into the potential of PEO-BG to accelerate bone repair. Additionally, long-term *in vivo* studies will be crucial to evaluating its implant survival and functional integration across diverse patient populations.

## 5 Conclusions

We successfully tested a bioactive glass-inspired coating for implants, developed using plasma electrolytic oxidation, in a preclinical model simulating diabetic patients with reduced bone density. Under these challenging circumstances, the PEO-BG coating enhances bone repair and osseointegration compared to conventional implant surfaces. Comprehensive assessments—including histological, histometric, micro-CT, immunohistochemical, and fluorochrome analyses—demonstrated the superior osteoconductive and osteoinductive performance of PEO-BG. The coating promoted early vascularization, cellular recruitment, bone matrix deposition, and the formation of a more organized and mature bone structure over time. Furthermore, we demonstrated the ability of PEO-BG to modulate the inflammatory response and upregulate key osteogenic markers, contributing to improved bone quality and repair. Given its biological efficacy, cost-effectiveness, ease of application, and long-term stability, the PEO-BG coating presents strong translational potential as an advanced implant surface modification, particularly for patients with systemic conditions that impair bone healing, such as diabetes mellitus.

## Supplementary Material



## Data Availability

The data presented in this study are available on request from the corresponding author. The data are not publicly available due to being part of the patent proposal.

## Notes

ARRIVE 2.0 guidelines (PDF)

## #

Department of Prosthodontics and Periodontology, Piracicaba Dental School, Universidade Estadual de Campinas (UNICAMP), Piracicaba, São Paulo, Brazil; Faculdade Israelita de Ciencias da Saude Albert Einstein, Hospital Israelita Albert Einstein, São Paulo 05652-900; Brazil
